# Multiple viral infections in *Agaricus bisporus* - Characterisation of 18 unique RNA viruses and 8 ORFans identified by deep sequencing

**DOI:** 10.1038/s41598-017-01592-9

**Published:** 2017-05-26

**Authors:** Gregory Deakin, Edward Dobbs, Julie M. Bennett, Ian M. Jones, Helen M. Grogan, Kerry S. Burton

**Affiliations:** 1 NIAB EMR, New Road, East Malling, Kent ME19 6BJ UK; 2 University of Reading, School of Biological Sciences, Reading, RG6 6UB UK; 3 Teagasc Food Research Centre, Ashtown, Dublin 15, D15 KN3K Ireland

## Abstract

Thirty unique non-host RNAs were sequenced in the cultivated fungus, *Agaricus bisporus*, comprising 18 viruses each encoding an RdRp domain with an additional 8 ORFans (non-host RNAs with no similarity to known sequences). Two viruses were multipartite with component RNAs showing correlative abundances and common 3′ motifs. The viruses, all positive sense single-stranded, were classified into diverse orders/families. Multiple infections of *Agaricus* may represent a diverse, dynamic and interactive viral ecosystem with sequence variability ranging over 2 orders of magnitude and evidence of recombination, horizontal gene transfer and variable fragment numbers. Large numbers of viral RNAs were detected in multiple *Agaricus* samples; up to 24 in samples symptomatic for disease and 8–17 in asymptomatic samples, suggesting adaptive strategies for co-existence. The viral composition of growing cultures was dynamic, with evidence of gains and losses depending on the environment and included new hypothetical viruses when compared with the current transcriptome and EST databases. As the non-cellular transmission of mycoviruses is rare, the founding infections may be ancient, preserved in wild *Agaricus* populations, which act as reservoirs for subsequent cell-to-cell infection when host populations are expanded massively through fungiculture.

## Introduction

Multiple viral infections occur in many Eukaryotic organisms, animals, plants and fungi. Seven RNA viruses have been sequenced from the grapevine, *Vitis vinifera*, associated with the disorder Syrrah/Shiraz decline^1, 2^ and multiple virus infections have been described in insects such as the honey bee^3, 4^. While in *Homo sapiens* infection with single viruses is mostly associated with a single pathogenic outcome, there is evidence that multiple viral infections can change disease symptoms^5–7^. In fungi, infection has been associated with a large numbers of distinct RNAs^8^: 26 RNAs have been identified in the fungus *Beauveria bassiana* with 11 in a single isolate^9^, and 26 RNAs found in the cultivated mushroom *Agaricus bisporus* with 16 in a single isolate^10^.

Multiple viral infections are often asymptomatic representing persistent static life-styles which appear to be benign, symbiotic or possibly beneficial to the host. For fungi, viral infection occurs predominantly via cytoplasmic exchange following cell-to-cell contact and *initial* infections from free non-cellular viruses have been difficult to demonstrate with only one example, a DNA virus, of fungal infection^8, 11^. Initial infections are therefore likely to be rare and ancient events which, once established, lead to long-term co-existence between virus and host in a non-lethal persistent life-style. Furthermore fungal growth, which is characterised by cytoplasmic exchange in the vegetative and sexual phases, promotes the accumulation of multiple viruses which then develop coexistence strategies within an individual hypha, colony or hyphal network.

Multiple virus infections involve numerous interactions among the viruses themselves, the host and the environment, resulting in changes at both the molecular and population levels that can lead to a transition in life-styles from persistent to acute, resulting in a disease phenotype^12^. Horizontal gene transfer has been inferred between positive-sense RNA viruses from different families in the fungal pathogen, *Sclerotinia sclerotiorum* ^13^. At the viral population level, Syller^14^ described different synergistic and antagonistic multiple viral interactions in plants leading to various outcomes, such as pathogenesis, viral evolution and the spatial separation of viruses among plant tissues. Host factors rather than viral titre or order of infection appear to have the biggest influence on the equilibrium levels of introduced viral strains to *Citrus* trees^15^. However, the concept of ‘quasispecies’ suggests that such equilibria can also be disrupted by changing environments. The quasispecies concept describes a “cloud” of viral sequences able to adapt by population and genetic change (mutation or selection of variants) leading to changes in viral-host interactions on the spectrum from symbiosis to pathogenicity^16–18^.

The fungus, *Agaricus bisporus*, whose genome was sequenced in 2012^19^, offers a model system for studying the interactions of multiple viral infections and transitions in viral life-styles. *A*. *bisporus* is commercially cultivated via vegetative propagation as a high value agricultural crop, the cultivated white mushroom, with an annual production value of $4.7bn^20^. *A*. *bisporus* can suffer from economically-damaging viral diseases such as La France disease and Mushroom Virus X (MVX) disease. La France disease has symptoms of growth retardation and distortion of fruitbodies and is associated with identifiable particles and a 9 segment virus, AbV1^21, 22^. MVX disease is a collective name for a mixture of symptoms (fruitbody browning, fruitbody retardation and distortion) associated with 30 dsRNAs, 26 found by Grogan *et al*.^10^, and an additional four by Eastwood *et al*.^23^. These RNAs are assumed to be unencapsidated viral genomes as no viral particles have been observed, although Romaine *et al*.^24^, found evidence of RNA virus packaging in membrane vesicles. Profoundly different levels of the low molecular weight RNA species (>10^3^ fold difference) have been observed in adjacent non-symptomatic and diseased *A*. *bisporus* fruitbodies attached to the same mycelial network, indicative of a spatially-separated, viral life-style transition, persistent to acute^23^.

The extent to which the sequences of the 30 viral RNAs of MVX are related, for example as defective or satellite RNAs, remains unknown. Two reports have presented evidence of hybridisation of two or more RNAs of the MVX complex with a single probe^23, 25^, while three viruses isolated from the Dutch Elm Disease fungus *Ophiostoma novo-ulmi* were found to be related^26^. Only two published RNA sequences have been associated with the MVX complex; the 14.5 kbp dsRNA identified as an Endornavirus^27^ and the partial sequence of a 17 kbp dsRNA hypothesised as a hypovirus^28^. Two further RNAs (1.8 and 2.0 kb) have been suggested to derive from a Partitivirus on the basis of their molecular size and apparent bipartite nature^23^.

Sequencing and phylogenetic classification of the multiple viruses associated with *A*. *bisporus* will enable a greater understanding of their relatedness, interactions and dynamics, host adaptations and viral life-style transitions. In this study 30 different viral RNAs from *A*. *bisporus* mushroom fruitbodies were sequenced, characterised and classified phylogenetically. To our knowledge this is the first *de novo* deep sequencing study of a large number virome for a single organism.

## Results

Ten dsRNA-enriched samples extracted from mushroom fruitbodies were sequenced and viral contigs were *de novo* assembled. Contig structures were confirmed by PCRs spanning the full length of the contigs and Sanger sequencing of select regions as well as RACE PCR and Sanger sequencing of the 5′ ends. These analyses revealed 30 distinct RNA molecules not found in the *A*. *bisporus* genome which ranged in size from 0.5 kb–14.5 kb (Table 1 and Supplementary Table S1). These sequences have been submitted to GenBank (accession numbers: KY357487 to KY357519, Supplementary Table S2) using the sequences present in sample 003 as reference where possible and sample 2990 for the remaining RNAs.

**Table 1:** Viral RNAs sequenced from samples of *A*. *bisporus* fruitbodies and their length and homology to known viruses.

Name	Short name		Contig	Length	Closest virus	Family/Genus	e-value
*Agaricus bisporus virus 2*	AbV2		C23-C1	14566	^†^ *Cryphonectria hypovirus 2*	*Hypoviridae*	7 × 10^−145^
*Agaricus bisporus endornavirus 1*	AbEV1		C40	12730	*Rhizoctonia cerealis endornavirus 1*	*Endornaviridae*	4 × 10^−98^
*Agaricus bisporus virus 3*	AbV3		C3	9340	*Cherry green ring mottle virus*	*Betaflexiviridae*	3 × 10^−28^
*Agaricus bisporus virus 5*	AbV5		C6	8371	*Citrus leaf blotch virus*	*Betaflexiviridae*	3 × 10^−50^
*Agaricus bisporus virus 6*	AbV6	RNA 1	C2	8848	*Cherry mottle leaf virus*	*Betaflexiviridae*	3 × 10^−30^
		RNA 2	C12	3559	^‡^ *Odontoglossum ringspot virus*	*Virgaviridae*	1 × 10^−28^
*Agaricus bisporus virus 7*	AbV7		C4	8759	*Botrytis virus F*	*Gammaflexiviridae*	6 × 10^−36^
*Agaricus bisporus spherical virus*	AbSV		C5	8540	*Cherry rusty mottle associated virus*	*Betaflexiviridae*	5 × 10^−31^
*Agaricus bisporus virus 8*	AbV8		C8	8280	*Beet necrotic yellow vein virus*	*Benyvirus*	2 × 10^−21^
*Agaricus bisporus virus 9*	AbV9		C13-C18-C14	7713	*Botrytis virus F*	*Gammaflexiviridae*	1 × 10^−27^
*Agaricus bisporus virus 10*	AbV10		C7	7033	*Fusarium graminearum dsRNA mycovirus-1*	^1^Unassigned	0
*Agaricus bisporus virus 11*	AbV11		C10	6981	*Fusarium graminearum dsRNA mycovirus-1*	^1^Unassigned	0
*Agaricus bisporus virus 12*	AbV12		C9	6893	*Oyster mushroom spherical virus*	^2^Unassigned	2 × 10^−41^
*Agaricus bisporus virus 13*	AbV13		C15	6202	*Rice stripe necrosis virus*	*Benyvirus*	7 × 10^−41^
*Mushroom bacilliform virus*	MBV		C11	4009	*Mushroom bacilliform virus*	*Barnaviridae*	0
*Agaricus bisporus mitovirus 1*	AbMV1		C41	3439	*Mitovirus AEF-2013*	*Mitovirus*	3 × 10^−65^
*Agaricus bisporus virus 14*	AbV14		C21	3482	*Epirus cherry virus*	*Ourmiavirus*	2 × 10^−3^
*Agaricus bisporus virus 15*	AbV15		C21a	3405	*Cassava virus C*	*Ourmiavirus*	5 × 10^−3^
*Agaricus bisporus virus 16*	AbV16	RNA 1	C22	1826	^‡^ *Broad bean mottle virus*	*Bromoviridae*	6 × 10^−81^
		RNA 2	C20	1949	^‡^ *Hydrangea chlorotic mottle virus*	*Betaflexiviridae*	9 × 10^−27^
		RNA 3	C29	781			
		RNA 4	C33	558			

The Name column corresponds to the proposed or given name for the discovered viruses. The Short Name column corresponds to a shortened version of the full name. The Contig column corresponds to contiguous RNA sequences assembled from the Illumina reads for each virus. The Length column is in RNA bases and corresponds to the total length of the virus. The Closest Virus column corresponds to the highest scoring homology (lowest e-value) to the given virus as identified with BLASTX. The e-value column indicates the likelihood of the BLASTX alignment occurring due to random chance. The Family/Genus column corresponds to the homologous viruses’ assigned taxonomic group.

^1^Proposed new members of the Hypoviridae family.

^2^Proposed new member of the *Tymovirales* order.

^†^The closest match was to a partial sequence of Agaricus bisporus virus X Acc.: CAD19173.

^‡^Alignments found using DELTA-BLAST.

**Figure 1:**
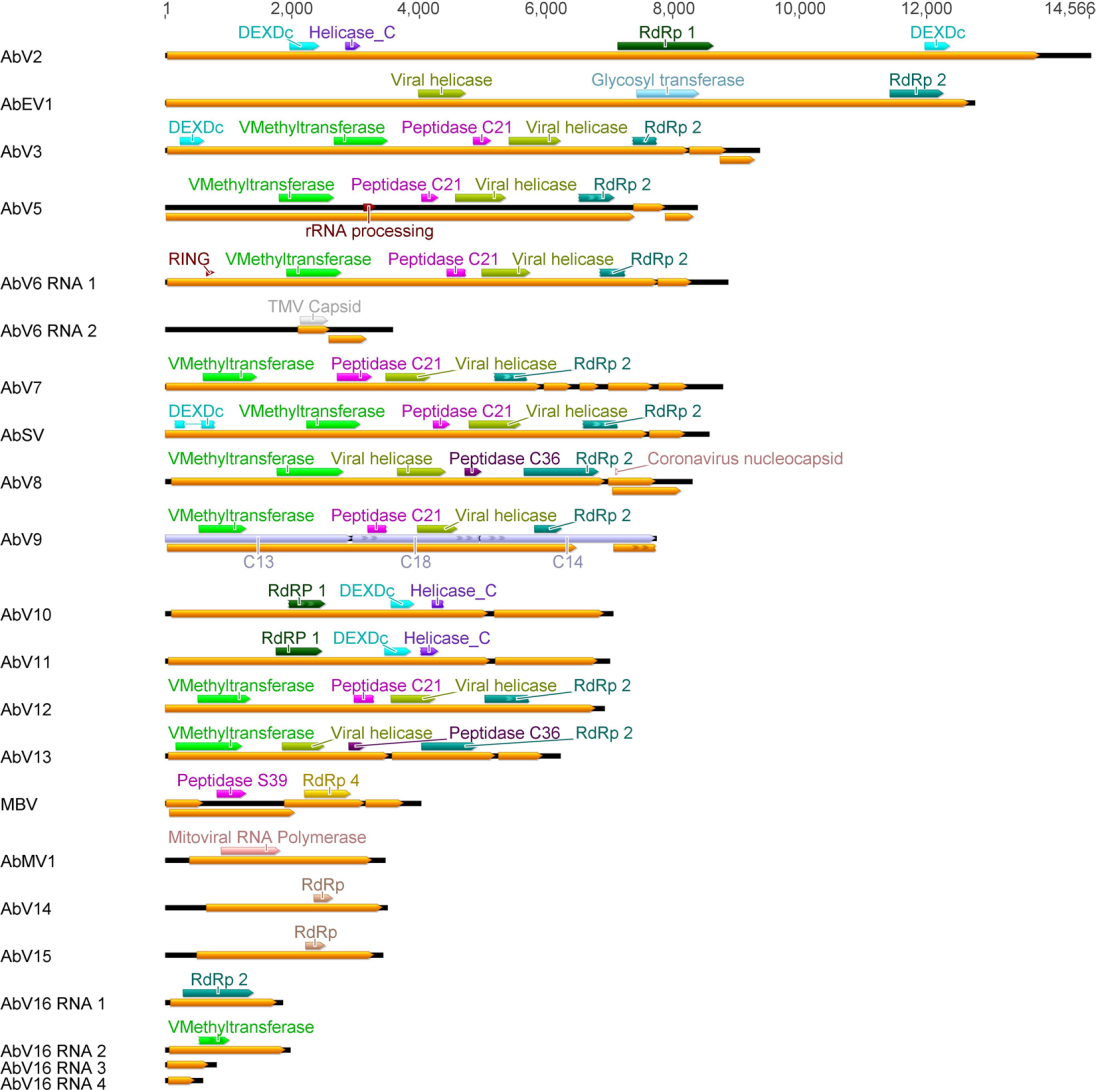
The genome organisation and protein coding potential of the viruses. ORFs greater than 300 bases on the + strand are shown as yellow annotations. Grey annotations indicate assembled contigs. Protein domains identified by NCBI Conserved Domain Search (E-value threshold 0.01) or HHPRED (p < 10–3) are shown as annotations in other colours. Incomplete annotations are shown with a jagged edge. Scale is length in bases.

Three of the 30 contigs have been previously described or sequenced: Mushroom Bacilliform Virus, MBV^29^, *Agaricus bisporus* Endornavirus 1, AbEV1^27^ and *Agaricus bisporus* Spherical Virus, AbSV (GenBank accession: AGH07918.1, 2013). Protein domains associated with RNA viruses of fungi and plants were found in 22 contigs (Fig. 1). Eighteen contigs contain an RNA dependant RNA polymerase (RdRp) domain (Fig. 1). Other identified viral protein domains coded for viral methyltransferases (Vmethyltransferase), helicases and capsid proteins (Fig. 1). A further 8 unique RNA molecules, all containing ORFs greater than 250 bases (described as ORFans 1–8) and lacking similarity to any known sequences, were also found (Supplementary Table S1).

We propose these RNA molecules represent 18 distinct viruses based on the presence of RdRp domains in each contig (Table 1). A nomenclature is proposed for these viruses of Agaricus bisporus Virus N (N replaced by a sequential number). This new nomenclature takes note of previous and existing descriptions made for *A*. *bisporus* viruses: AbV1 – the causative agent of La France disease^21^ and AbV4^30^ and so starts at AbV2, AbV3, and continues with AbV5. In addition, a mitochondrial virus was identified and named AbMV1 (*Agaricus bisporus* Mitochondrial Virus 1).

Sixteen of the contigs were consistent with monopartite viruses. In addition we hypothesise two, novel, segmented viruses^1^: AbV6 consisting of AbV6 RNA 1 (C2) with an RdRp domain and AbV6 RNA 2 (C12) with a capsid-like domain with homology to Tobacco Mosaic Virus (TMV); and^2^ AbV16 consisting of four separate contigs, AbV16 RNA 1 (C22) containing an RdRp domain, AbV16 RNA 2 (C20) containing a Vmethyltransferase, and AbV16 RNA 3 (C29) and AbV16 RNA4 (C33) each with open reading frames of unknown function. Both viruses contained shared motifs in their 3′ UTRs, no 5′ UTRs shared motifs were found in any of the sequenced RNAs.

We have assigned C13, C14 and C18 to represent a single viral molecule, AbV9, however the evidence for this is incomplete and it may represent a segmented virus. C13, C14 and C18 contained different domains (Vmethytranserase, peptidase, RdRp and viral helicase) (Fig. 1), were of similar GC content (58.2%, 58.4%, 59.4% respectively), and their abundances were tightly correlated (C13 vs C14 r = 0.99, C13 vs C18 r = 0.99, C14 vs C18 r = 0.99). As a separate molecule C13 did not contain an in-frame stop codon, and the larger hypothetical construct encodes a single ORF contiguous across all three contigs which encodes all four viral domains arranged in the same order as found in the Tymoviridae family. Assembly of C13, C14 and C18 consistently produced three separate contigs and the conjoining ends of each separate contig contained mononucleotide runs. We were able to PCR across the hypothetical construct albeit resulting in PCR products of multiple sizes at the contig junctions (not shown). The rich GC contents of the three contigs may have confounded assembly programs and/or inhibited PCR efficiency.

The abundance of the viruses and ORFans, determined as the number of fragments sequenced per kb of contig, varied widely among samples (Table 2). The four AbV16 RNAs were found at high levels in the samples showing overt disease (003, 004 and 1497) but absent from the remainder. AbV6 RNAs were present at high levels in 7 of the samples while AbV2, AbV6, AbV10 and ORFans 2, 3, 4, 5 and 7 were found in all ten samples. AbV15, AbMV1 and ORFan1 were found in only two samples (Table 2).

**Table 2:** Abundance (depth of sequencing coverage) of viral RNAs and ORFans for each sample, determined as number of fragments per kb of transcript per million mapped reads.

Virus		Sample Number									
		138	003	004	1497	3209	1283	2735	2786	2919	2990
AbV2		314	2536	1562	1939	168	53	295	77	29	42
AbEV1		3	0	0	0	2	4805	2	1	2	15177
AbV3		2	3678	2249	1	3	2	3	2	31297	3
AbV5		0	4344	2	1	0	0	0	0	0	0
AbV6	RNA1	4	4482	3138	2135	3	18234	264	14409	7321	3822
	RNA2	11	16092	8507	1487	12	42093	146	53598	21323	40421
^†^AbV6–2990		0	0	0	0	0	0	0	0	0	7349
AbV7		1	5378	956	2956	1	14	817	31	24	19
AbSV		1	190	397	68	1	0	35	274	58	53
AbV8		1	1	1	2580	1	384	1	4382	1	88
AbV9		0	417	178	1	0	0	0	0	0	0
AbV10		130	1794	1164	501	260	2336	1703	4216	288	1132
AbV11		1	2348	868	566	220	519	1	1153	561	135
AbV12		0	297	83	318	69	22	0	68	7	4
AbV13		0	776	1	0	0	39	0	0	0	45
MBV		1	1318	569	956	0	0	0	0	0	2
AbMV1		0	0	0	0	0	774	0	1	0	66
AbV14		1	71	2	63	0	615	0	741	387	0
AbV15		0	81	1	0	0	0	0	0	0	357
AbV16	RNA1	3	9116	7438	16157	0	0	0	1	0	0
	RNA2	2	10679	7405	19814	0	0	0	0	0	0
	RNA3	4	14426	10008	27894	0	0	0	0	0	0
	RNA4	10	17781	17707	47372	0	0	0	0	0	0
ORFan 1		0	9	24	0	0	0	0	0	0	0
ORFan 2		172	7232	5570	3295	143	378	83	727	178	281
ORFan 3		164	1160	708	1111	63	21	135	26	8	15
ORFan 4		74	121	84	686	66	908	74	2000	153	256
ORFan 5		18	200	82	99	21	111	17	235	40	59
ORFan 6		0	72	3	0	0	0	0	0	0	0
ORFan 7		116	1286	851	2244	201	314	95	864	43	110
ORFan 8		0	7180	2900	9678	0	0	0	0	0	0

^†^AbV6–2990 is a strain of AbV6 RNA1 found in sample 2990.

The RdRps of the 18 viruses have closest amino acid homology to diverse positive sense single-stranded RNA (ss(+)RNA) viral orders/families/genera: *Hypoviridae*, *Tymovirales* (*Betaflexiviridae* and *Gammaflexiviridae)*, *Narnaviridae*, *Barnaviridae*, *Bromoviridae*, *Endornaviridae*, *Virgaviridae*, the unassigned genera, *Benyvirus* and *Ourmiavirus* and a proposed new family, *Ambsetviridae* (below).

However, phylogenetic analysis based on comparisons of RdRp sequences with known viruses identified only two viruses that could be readily assigned to known viral families, and a further two formed a clade with a single unassigned hypovirus-like virus. The majority are only distantly related to known viruses, and accordingly new phylogenies are proposed (Supplementary Figures S1, S2, S3, S4 and S5). A search of the Expressed Sequence Tag (EST) and Transcriptome Shotgun Assembly (TSA) databases revealed new hypothetical viral sequences which were also included into the phylogenetic analyses (Table 2, Supplementary Figures S2, S3 and S5 and Supplementary Tables S8 and S9).

### *Hypovirus* like sequences

Three viruses were identified with significant similarity to members of the *Hypoviridae* family, AbV2 (14.6 kb), AbV10 (7.03 kb) and AbV11 (6.98 kb) (Table 1). AbV2 encompassed the partial sequence previously identified by Sonnenberg and Lavrijssen^28^ and had 28% sequence identity at the protein level to *Cryphonectria hypovirus 2* (CHPV2).

**Figure 2:**
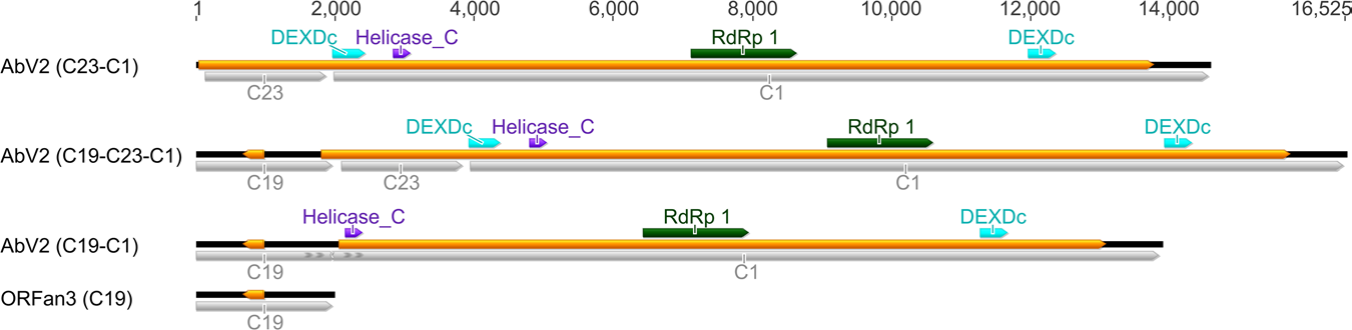
Inferred genome structure of the three AbV2 modular variants, AbV2 (C23-C1), AbV2 (C19-C23-C1) and AbV2 (C19-C1), and the associated ORFan ORFan3 (C19). Predicted ORFs greater than 300 bases are shown as yellow annotations. Grey annotations indicate size and position of sequenced contigs. Scale is length in bases.

Several different variants of AbV2 were detected differing in their 5′ end sequences (Fig. 2 and Supplementary Table S3). We define the standard version of AbV2 as C23-C1 based on the prevalence of its constituent contigs (Supplementary Table S3). Some assemblies identified ORFan3 (C19) to be joined to AbV2. A longer variant of AbV2 (C19-C23-C1) was found in the assemblies in which ORFan3 was fused to its 5′ end and this was confirmed by PCR across the junction. A shorter variant, AbV2 (C19-C1) was assembled and confirmed by Sanger sequencing, where ORFan3 replaced a 2654 nucleotide section at the 5′ terminus of AbV2. This variant had a shorter ORF of 11 kb and lacked the N-terminal DEXDc helicase domain.

AbV2 variants C23-C1 and C19-C23-C1 contained a C terminal RdRp and helicase similar to other hypoviruses^31^ (Fig. 2) as well as an atypical second DEXDc helicase domain near the N terminus which was more similar to helicases found in viruses of the *Potyviridae* family than other Hypoviruses (Fig. 2). No DEXDc helicase domains have been found in any other members of this order and the result is suggestive of horizontal gene transfer.

AbV10 and AbV11 have amino acid similarity to members of the *Hypoviridae* family, however they were more closely related (by nucleotide alignment) to the unassigned *Fusarium graminearum* dsRNA Mycovirus-1 (FgVDM1). The inclusion of AbV2, AbV10 and AbV11 in phylogenetic analyses enabled an enhanced classification of the *Hypoviridae* (Supplementary Figure S1 and Supplementary Table S4).

### *Narnavirus* like sequences

Three novel viruses were identified as putative members of the *Narnaviridae* family (AbV14, AbV15 and AbMV1). The *Narnaviridae* are amongst the simplest of RNA viruses consisting of a single, short replicase polyprotein (2.3–3.6 kb). There are two genera within the family, the *Mitovirus* and *Narnavirus*, with different subcellular locations, mitochondrial and cytosol respectively^32^.

AbMV1 had closest homology to Mitovirus AEF-2013, a presumed fungal mitovirus isolated from a dipteran fly species^33^ (Table 1). The AbMV1 genome comprised a single ORF with a mitoviral RNA polymerase domain, translated using the mitochondrial code (Fig. 1) and had a GC content of 35% (Supplementary Table S5) similar to that of the mitochondrial genome (*A*. *bisporus* GC% is 46.2% for nuclear DNA and 28.6% for mitochondrial DNA^19^).

AbV14 and AbV15 were closely related with 57.7% amino-acid identity. The phylogeny of the *Narnaviridae* has been revised by the inclusion of three new *A*. *bisporus* viruses (AbV14, AbV15 and AbMV1), six hypothetical viruses identified here from the TSA databases and six more described by Cook *et al*.^33^ (Supplementary Figure S2 and Supplementary Table S6).

### *Tymovirales* like sequences

Six viruses had closest homology to members of the *Tymovirales* order (*Betaflexiviridae* and *Gammaflexiviridae*), AbV3, AbV5, AbV6, AbV7, AbV9 and AbSV (Table 1). In addition AbV12 appears to be related to the *Tymovirales* order with closest similarity (using BLASTX) to the unassigned ss(+)RNA virus OMSV, Oyster mushroom spherical virus (Supplementary Figure S3).

The Tymovirales are monopartite ss(+)RNA viruses infecting plants and fungi with genomes of 5.4 kb–9 kb, the structure of which places them within the alpha-like super group of viruses.

The seven *A*. *bisporus* viruses in the *Tymovirales* have large replication ORFs of size range 5.9 kb–8.2 kb, with each containing Vmethyltransferase, protease C21, viral helicase and type II RdRp domains characteristic of members of the alpha-like supergroup. A majority also contained additional domains (Fig. 1). A new phylogeny of the Tymovirales is proposed by analysis of the RdRp domains of these seven viruses and two hypothetical viruses identified in the TSA database (from *Agave tequilana*: accessions GAHU01087027 and GAHU01094215) with high similarity to the C14 component (RdRp) of AbV9). This places the viruses into three new clades within the *Tymovirales* order, tentatively named *Readiviridae*, *Emraviridae*, *Teagaviridae* (Supplementary Figure S3).

**Figure 3:**
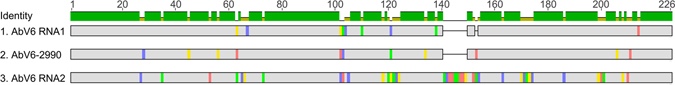
Alignment of the final 226 bases of the 3′ UTR of AbV6 RNA1, AbV6-2990 (a strain of AbV6 found in sample 2990) and AbV6 RNA2. The grey regions indicate bases that match the consensus sequence (defined as sequence shared by two out of 3 of the RNAs) whilst the coloured highlights indicate disagreements. Scale is length in bases.

We propose AbV6 is a bipartite segmented virus consisting of the AbV6 RNA 1, encoding a replicase domain, and AbV6 RNA 2 encoding a capsid- like motif. The abundance of AbV6 RNA1 and AbV6 RNA2 across all 10 samples is highly correlated (*r* = 0.95) (Table 2). A distinct strain of AbV6 RNA1 (AbV6-2990), 8921b in length, was identified in sample 2990 with 88% pairwise identity with AbV6 RNA1 and with the same genome organisation. A 226 base motif was identified in the 3′ UTRs of AbV6 RNA1, strain AbV6-2990 and AbV6 RNA2 (Fig. 3).

AbV6 and strain AbV6-2990 both contain zinc-finger RING domains (Pfam 13920) at the N terminus. AbV3 and AbSV contained a tymovirus cysteine endopeptidase as well as an N terminal DEXDc helicase while AbV5 had a domain similar to a ribosomal RNA processing protein (PF08524).

### *Benyvirus* like sequences

AbV8 and AbV13 have sequence similarity to the replicase segment of the *Benyvirus* genus within the Rubi-like virus grouping (Table 1 and Supplementary Figure 4). Of the two ORFs in AbV8, the larger contained Vmethyltransferase, helicase, protease and RdRp domains (Fig. 1) while the second ORF was of unknown function, although it contained a nucleocapsid motif as predicted by HHpred (e-val = 2.6E-05, p = 1.6E-09). AbV13 had a different genome coding strategy with three ORFs; ORF1 contained a helicase domain, ORF2 contained an RdRp and ORF3 coded for a protein of unknown function (Fig. 1).

### AbV16 – the proposed causative agent for the Brown Cap Mushroom Disease

**Figure 4:**
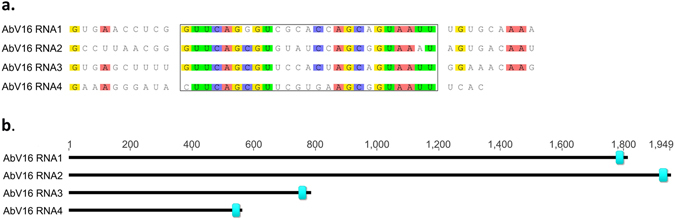
The motif sequence and location shared by the AbV16 RNAs (**a**) Alignment of the motif observed in the 3′ UTR of the AbV16 RNAs which are associated with the Brown Cap Mushroom Disease. The nucleotides within the box define the shared motif with probabilities of; 4.87 × 10^−13^, 1.0 × 10^−12^, 1.36 × 10^−14^ and 1.07 × 10^−12^ for RNAs 1–4 respectively. (**b**) The location of the motif within each RNA molecule. Scale is length in bases.

We propose AbV16 to be a segmented virus consisting of four components: AbV16 RNA1, encoding a type II RdRp, AbV16 RNA2, encoding a type 1 Vmethyltransferase domain and AbV16 RNA3 and AbV16 RNA4 each of which encodes an ORF with no homology to known sequences (Fig. 1). All four molecules were found at high abundance in the three samples showing fruitbody browning symptoms (Table 2). In addition, all of the AbV16 RNAs contained a 25 base motif in the 3′ UTR, identified using MEME^34^ (Fig. 4). The motif: “STTCAGSGTBBVWSHAGCRGTAAWT” had a probability of 8.2 × 10^−4^, and was the only motif found in AbV16 RNAs with a probability less than 1. The four AbV16 molecules and ORFan8 were homologous to the partial viral transcripts previously associated with browning symptoms reported by Eastwood *et al*.^23^ (Supplementary Table S7).

Initial phylogenetic analysis of the RdRp domain of AbV16 RNA 1 was unable to place it consistently within any clades of the alphavirus-like supergroup. A BLASTP search of the TSA and EST databases using the translated ORFs revealed sixteen sequences with high similarity to AbV16 RNA 1 (Table 3). Nine of these datasets also contained homologs to AbV16 RNA 2 while two contained homologs to AbV16 RNA 3 (Table 3). Phylogenetic analysis of the RdRp domains from viruses in the alphavirus-like supergroup, AbV16 RNA 1 and its homologs from the TSA and EST database searches (Table 3) placed AbV16 and its homologs into their own unique clade (Supplementary Figure S5) distinct from all previously described viral groupings. Therefore, the hypothesis is made for a new viral family which has been named *Ambsetviridae* (after the births Amber Deakin and Seth Dobbs during the course of this research).

**Table 3:** Homologs and proposed members of the *Ambsetviridae* family of AbV16 with an e-value < 10^−3^ identified in NCBI Expressed Sequence Tag and Transcriptome Shotgun Assembly databases with BLASTP.

Host	Accession(s)	AbV16	e-value
^1^ *Populus tremula* × *P*. *tremuloides*/*Amanita muscaria*	*AJ642818, AJ643643, AJ645918, AJ642922	RNA 1	1 × 10^−103^
	AJ643396, AJ646169, AJ641181, AJ646464	RNA 2	2 × 10^−41^
	AJ645442	RNA 3	1 × 10^−4^
^1^uncultured eukaryote (Paal)	*F0138635	RNA 1	1 × 10^−25^
	FO138385	RNA 2	4 × 10^−16^
	FO146723	RNA 3	9 × 10^−4^
^1^uncultured eukaryote (Balen)	FO124478	RNA 1	2 × 10^−25^
	FO119421	RNA 2	2 × 10^−17^
^*1*^ *Avena barbata*	*GR358402	RNA 1	8 × 10^−48^
	GR348442	RNA 2	2 × 10^−16^
^*1*^ *Festuca pratensis*	*GO798150	RNA 1	1 × 10^−69^
	GO893996	RNA 2	4 × 10^−31^
^*2*^ *Hevea brasiliensis*	*JR365510	RNA 1	7 × 10^−23^
	JR348216	RNA 2	5 × 10^−4^
^*1*,*2*^ *Humulus lupulus*	*GAAW01004482, GAAW01052131	RNA 1	1 × 10^−36^
	GD247813, GAAW01040225	RNA 2	9 × 10^−12^
^*2*^ *Sesamum indicum*	*JP662946	RNA 1	2 × 10^−57^
	JP662731 & JP660300	RNA 2	1 × 10^−16^
^*2*^ *Cercis gigantea*	GAOK01000859	RNA 1	1 × 10^−68^
	GAOK01003225	RNA 2	3 × 10^−9^
^*1*^ *Cryptomeria japonica*	*BY888444	RNA 1	1 × 10^−21^
^*1*^ *Picea glauca*	*EX369669	RNA 1	1 × 10^−36^
^1^uncultured eukaryote (Paal)	FO147196	RNA 1	4 × 10^−23^
^*1*^ *Deschampsia antarctica*	FL686666	RNA 1	4 × 10^−57^
^*2*^ *Elaeis guineensis*	*GAJH01044042	RNA 1	6 × 10^−80^
^*2*^ *Pachycladon fastigiatum*	JR014949	RNA 1	8 × 10^−11^
^2^ *Pseudomallada prasinus*	GAVV01159677	RNA 1	2 × 10^−30^
^*1*^ *Rosa virginiana*	JZ193315	RNA 2	1 × 10^−16^
^1^Saccharum hybrid cultivar	CA278838	RNA 2	4 × 10^−15^

The Host column corresponds to the organism (if known) from which RNA was extracted. The Accession column corresponds to the GenBank accession(s) for the homologous sequence. The AbV16 column lists the AbV16 RNA to which the homolog was found and the e-value column lists the BLASTN e-value.

^*^Indicates used in construction of phylogenetic tree (Supplementary Figure S5).

^1^Sequence found in Expressed Sequence Tag database.

^2^Sequence found in Transcriptome Shotgun Assembly database.

### ORFans

We identified eight further non-host RNAs ranging in size from 0.5 kb to 5 kb with potential open reading frames of greater than 250 bases (Supplementary Table S1). These sequences were named as ORFans rather than satellites as they lacked any 5′ or 3′ UTR homology to the viruses present and the potential ORFs lacked similarity to any known sequence. ORFans 4, 5, and 7 each consisted of several repeating regions and may be circular and potentially code for proteins of unknown function. ORFans 2, 3, 4, 5, and 7 were found in all samples. ORFan 1 and ORFan6 were found in samples 003 and 004 and then only in low copy number.

ORFan 8 has sequence homologous to the RNA molecules VX7 (28; and Anton Sonnenberg: personal communication) and a transcript fragment identified by Eastwood *et al*.^23^ associated with the fruitbody browning (Supplementary Table S7). Although ORFan8 had similar levels of abundance to the AbV16 RNAs (Table 2), it was not classified as a component of AbV16 virus as it lacked the 3′ motif.

## Discussion

Multiple virus infections have been identified in mushroom fruitbodies of *Agaricus bisporus* by the discovery of 18 RNA viruses and 8 ORFans by deep sequencing. The viruses are phylogenetically distinct, the majority with low similarity to any virus previously described and were neither satellite nor defective RNAs. They represent the most extensive collection of unrelated multiple viral infections yet described, the closest comparable example being for a single diseased isolate of *Ophiostoma novo-ulmi* which contained 12 viral RNAs, all related mitochondrial viruses^32^.

Despite the multiple viral infections, the *A*. *bisporus* cultures grew and thrived, extracting nutrients from a complex substrate and producing fruitbodies, suggesting effective co-existence strategies and adaptive mechanisms. Samples 003, 004 and 1497 displayed only mildly debilitating (although economically important) symptoms of fruitbody browning while harbouring 17–24 viral RNAs (viruses and ORFans) (Table 3). Similarly, the 10–17 viral RNAs identified in fruitbodies grown from sub-cultures, and the 8 viral RNAs in commercial culture (Table 2), are characteristic of viruses with persistent life-styles and low fitness costs on the host^35^. The *A*. *bisporus* genome contains the components of viral defence mechanisms with homologs to both RNAi and yeast SuperKiller (SKI) mechanisms^19^ and RNAi has been demonstrated in *A*. *bisporus* ^36–38^. It is possible that some ORFs encode viral suppressors of RNAi but the identity and nature of any suppressors were not revealed by this research. However, the viral genomes contained many peptidases, numerous ORFs of unknown function and several domains of cryptic function (e.g. RING domain on AbV6 and rRNA processing on AbV5).

The 18 viruses have the closest amino acid homology to the RdRp of single-stranded RNA viruses. The assignment of ‘single-strandedness’ is at variance with both previous descriptions of the *A*. *bisporus* RNAs as double-stranded^10, 25, 28^ and the intended specificity of the purification procedures. It is possible therefore that these viruses have secondary and tertiary structures that enable selective binding to cellulose and protection from S1 nuclease. Three of the viruses have domains with similarities to capsid proteins (AbV6 RNA2, MBV and AbV8), although no viral particles have been observed in MVX-infected tissues^10^. Two of the viruses are hypothesized to be multipartite (AbV6 and AbV16) on the basis of correlative abundance in different samples and a common 3′ motif.

The extent of the multiple infections reported here is suggestive of a diverse, dynamic and interactive ecosystem with evidence of sequence variation, alternative splicing, horizontal gene transfer, and variable fragment number (AbV16). Sequence variability ranged from 0.04–0.07 SNP’s/kb (for ORFan2, ORFan3 and AbV5) suggesting a high fidelity replicase to two orders of magnitude higher for AbV16 RNA1, AbV14 and AbV16 RNA2 (8.9–10.1 SNP’s/kb) (Supplementary Table S5). AbV2 displayed alternative splicing of C23, C19 and C19-C23 fused at the 5′ terminus and 2.6 kb from the 5′ terminus of C1. The C1 component of AbV2 contains different helicase domains from phylogenetically distinct orders/families suggestive of horizontal gene transfer (Fig. 2 and Supplementary Figure S1b). Evidence supporting horizontal gene transfer has also been found between distinct viruses in *Sclerotinia sclerotiorum* ^13^ and between fungal species^39^. In this respect, the composition and abundance of *A*. *bisporus* viruses appears to be similar to that of plants and plant viruses, showing infection gains from the environment and losses probably due to competition and antagonistic interactions^14^. Local infection is inferred for sample 003 as both samples 003 and 004 were grown from *A*. *bisporus* inoculated compost from the same source, their fruitbodies collected on the same day, yet 003 had two additional viruses (AbV13 and AbV15) (Table 2). Virus loss was indicated for sample 2735, as it had high abundance of AbV16 RNAs when first collected (data not shown) but no AbV16 RNAs detected in this study. Similarly, loss of AbV16 RNA1 has been demonstrated over 8 days compost culture between the first and second flush of fruitbody production (AbV16 RNA1 described as the 1.8 kb RNA or band 19 in ref. 40). The mechanism for the loss of AbV16 RNAs may be related to the large changes in levels of AbV16 detected during viral life-style transitions, persistent to acute, in individual fruitbodies^23^.

The *Ambsetviridae* proposed as a new viral family of plant and fungal viruses comprised the four component virus AbV16 and homologues found in the TSA and EST databases (Table 3). The common 3′ motif in the four RNAs of AbV16 may represent part of a mechanism for co-ordinated replication. However, a further RNA molecule, ORFan8, was also tightly correlated in abundance with AbV16 RNAs yet lacked the 3′ motif (Table 2). AbV16 may thus have a variable number of fragments: AbV16 RNAs 1–4, with replication controlled by the common 3′ motif and with ORFan8, whose co-replication is determined by a different mechanism. The apparent discrepancy of 4 or 5 RNAs associated with the browning symptom has been previously reported by Grogan *et al*.^10^ who identified 4 RNAs from UK samples while Sonnenberg and Lavrijsen^28^ described 5 RNAs in samples from The Netherlands. The four components of AbV16 and ORFan8 encompass the transcript fragment sequences identified by Eastwood *et al*.^23^ as being associated with a persistent/acute life-style transition (genome copies varying by 10^3^ fold) and likely to be the aetiological agent of Brown Cap Mushroom Disease. The high variability found in AbV16 RNAs 1 and 2 is consistent with the high mutation rates associated with acute viruses^35^.

The fruitbodies grown from a non-diseased commercial culture (sample 138) also harboured ubiquitous viruses and ORFans (i.e. AbV2, AbV6, AbV10, ORFan2, ORFan3, ORFan4, ORFan5 and ORFan7). Two of these show high similarity to those previously reported (by sequence, size and presence in non-symptomatic fruitbodies): the AbV2 sequence corresponded to the 14–17 kb RNAs of Sonnenberg and Lavrijsen^28^, the 16.2 kbp RNA of Grogan *et al*.^10^ and the >13 kbp L-RNAs of Kuang *et al*.^25^; and ORFan2 is likely to be the 2.7 kb VX3 of Sonnenberg and Lavrijsen^28^, the2.4 kb S-RNA of Kuang *et al*.^25^ and the 2.4 kb RNA of Grogan *et al*.^10^ who identified this RNA in 99% of the 320 healthy or diseased mushroom samples tested. These ubiquitous and persistent viruses have clearly developed efficient strategies for replication^12, 35^ which may have been amplified by cultivation and breeding of the host.

Geographical differences in the viral sequences were identified. The sequence of the North American ORFan2 (ref. 25 and pers. com. Peter Romaine) had 106 bases different at the 3′ end from the European ORFan2. In addition strain AbV6–2990 was found only in the sample from the Middle East while the related strain AbV6 was found in both Middle Eastern and European samples. These differences may relate to geographically distinct sequence variants or to differences in local reservoirs of infection.

Mycoviruses are generally considered to lack infectivity as extracellular free particles however one report demonstrated infection by a purified DNA mycovirus^11^. This lack of infectivity suggests that the original infections of *A*. *bisporus* may be ancient, before widespread cultivation in the 17^th^ century^41^ and probably represent numerous, separate infection events. The cultivation of *A*. *bisporus* is an international business involving long-distance movement of commercial cultures, colonised compost and mushroom fruitbodies, all of which are likely to increase the reservoirs of infected material and enhance virus dispersal leading to co-infections by viruses previously separated geographically. Virus transmission from wild to cultivated *A*. *bisporus* cultures probably occurred via aerially dispersed hyphae and spores. The longevity of basidiomycete fungi in the wild^42^, their propensity for vegetative growth and endurance would naturally facilitate the long-term survival of reservoirs of virus-infected mycelium in the environment.

Improved sequencing of host and virus genomes and bioinformatic techniques have suggested that multiple virus infections of eukaryotes may be more common than previously indicated by the classical application of Koch’s postulates. In this case disease may be the consequence of an altered balance among members rather than the presence of one sequence *per se*. In the apathogenic state, interactions between host and viruses involve complex co-existence strategies for symbioses. However, a changed environment can lead one virus such as AbV16 to replicate to very high levels and become pathogenic. Our data suggest that diagnostic tests for mushroom browning may need to consider relative virus load as well as the presence or absence of critical RNAs. Furthermore, the potential exists to re-balance virus numbers in favour of apathogenicity if the environmental factors leading to selective amplification and transmission through mycelial networks can be understood. This will be the subject of further reports.

## Materials and Methods

### Biological material and sample collection

Next Generation Sequencing was performed on RNA extracted from ten samples of *Agaricus bisporus* mushroom fruitbodies (Table 4), all strain A15 (Sylvan: www.sylvaninc.com). Three of the samples consisted of diseased mushrooms (displaying the fruitbody browning symptom) collected from different mushroom farms in 2004 and 2011 (Table 2). Six further samples were from fruitbodies grown at Kinsealy Research Centre, Ireland in simulated commercial conditions of mycelial sub-cultures taken from diseased fruitbodies (displaying a range of viral disease symptoms) collected between 2000–2004 and maintained in the laboratory as mycelial sub-cultures as per Grogan *et al*.^43^ (Table 4). Fruitbodies from the sub-cultures did not display the brown tissue symptom. In addition fruitbodies were grown and collected (sample 138) from non-diseased commercial cultures (Sylvan Inc. Ireland) at Kinsealy Research Centre, Ireland. All fruitbodies were frozen in liquid nitrogen and stored at −80 °C until use.

**Table 4:** Details of samples used for Next Generation Sequencing.

Code number for *A*. *bisporus* samples	Date of disease outbreak	Industry sample or cultured at Teagasc	Country of origin	Sample collected or produced by:
003	11/2011	Industry	Rep of Ireland	Teagasc
004	11/2011	Industry	Rep of Ireland	Teagasc
1497	09/2004	Industry	Belgium	Wageningen UR
1283	02/2000	Cultured	England, UK	Teagasc
2735	02/2002	Cultured	Rep of Ireland	Teagasc
2786	03/2002	Cultured	Netherlands	Teagasc
2919	06/2002	Cultured	England, UK	Teagasc
2990	08/2002	Cultured	Middle East	Teagasc
3209	01/2003	Cultured	England, UK	Teagasc
138: Non-diseased commercial culture	N/A	Cultured	Rep of Ireland	Teagasc

### RNA extraction from mushroom fruitbodies

Fruitbodies were freeze dried and ground in liquid nitrogen. 3.5 g dry weight of each ground mushroom sample was suspended in 4x w/v of an STE-based lysis buffer, pH7, and RNA was extracted with an equal volume of 5:1 phenol chloroform (pH 4.5). The RNA was purified and enriched for dsRNA by the addition of ethanol to 16%, followed by chromatography through two cellulose columns (medium fiber cellulose – Sigma-Aldrich, Dorset, UK), using a method modified from the protocol of Morris and Dodds^44^. 15–20 extractions from each fruitbody sample were combined before digestion of DNA and ssRNA by DNAse I and S1 Nuclease (Thermo Scientific, Waltham, UK) respectively.

### Quality control of purified RNA Extracts

The purity of RNA extracts was assessed by absorption spectra using a Nano-drop 1000 (Thermo Scientific, Waltham, UK) and the level of degradation was assessed using size separation by capillary electrophoresis on a Fragment Analyser (AATI, Ankeny, USA). Prior to sequencing RNA samples were quantified using the Qubit RNA BR Assay (Invitrogen, Paisley, UK) and assessed for quality on a R6K Tapestation (Agilent Technologies, Santa Clara, USA).

### Next Generation Sequencing

cDNA and library construction were performed using NEBNext mRNA Sample Prep Master Mix Set 1 (New England Biolabs Inc, Ipswich, USA) according to manufacturer specifications, with the following details: ~100 ng total RNA used per sample, no polyA or ribosomal depletion, bead size selection 200–600 bp and 12 cycles PCR. For samples 003, 004 and 1497, libraries were constructed using two different denaturation conditions (1) 72 °C for 3 mins or (2) 95 °C for 10 mins. For the remaining samples the denaturation conditions were 95 °C for 10 mins.

Adapters were ligated using the Multiplexing Sample Preparation Oliogonucleotide Kit (Illumina Inc, San Diego, USA) and each library was size selected using Ampure XP beads (Beckman Coulter UK Ltd, High Wycombe, UK). The following custom primers (25 µM each) were used for the PCR enrichment step: (1) Multiplex PCR primer; 1.0 5′-AATGATACGGCGACCACCGAGATCTACACTCTTTCCCTACACGACGCTCTTCCGATCT-3′, and (2) Index primer; 5′-CAAGCAGAAGACGGCATACGAGAT[INDEX*]CAGTGACTGGAGTTCAGACGTGTGCTCTTCCGATCT-3′, *The index refers to eight base tags developed by Wellcome Trust Centre for Human Genetics (Oxford, UK).

The amplified library was purified using Ampure beads (Beckman Coulter UK Ltd, High Wycombe, UK) and size distribution determined using a Tapestation D1K system (Agilent Technologies, Santa Clara, USA). Libraries were quantified by Picogreen (Invitrogen, Paisley, UK) and pooled to provide equal quantities of RNA per library.

Finally, a Quantitative PCR was performed, using Agilent qPCR Library Quantification Kit and a MX3005P instrument (Agilent Technologies, Santa Clara, USA), to measure the relative concentration of the pool compared to a previously sequenced mRNA library in order to determine the volume to use for sequencing. Sequencing was performed on an Illumina MiSeq with a read length of 150 bp, paired end reads and an average insert size of ~200 bp, at the Wellcome Trust Centre for Human Genetics (Oxford, UK).

### Data filtering

Fastqc was used to check the quality of the read data. Reads were trimmed using Trimmomatic to remove nucleotides with quality less than Phred33+15 and to only retain reads of 35 nucleotides or greater in length. Bowtie was used to align reads to *Agaricus bisporus* genes, known rRNA, *Pseudomonas* and PhiX and these reads were excluded from the assembly.

### Assembly

*De novo* assembly was carried out using Velvet v1.2.08 and redundant contigs were removed using Oases v0.2.08 and the Fastx toolkit. Cap3 was used to assemble the smaller contigs into larger scaffolds, producing 39–140 contigs per sample.

### Virus discovery pipeline

The NCBI Batch Web CD-Search Tool (default settings) was used to search the assembled contigs for conserved protein domains. Based on the domains returned contigs were assigned to one of four groups; (1) viral, (2) non-viral, (3) chimera (i.e. mix of viral and non-viral) or (4) unknown (if no domains found). Contigs which were deemed to be of non-viral or chimeral origin were discarded.

Open Reading Frames (ORFs) and the flanking Untranslated Regions (UTRs) were predicted using Geneious (6.0.6)^45^ as well as the GC content for each of the contigs. The ORFs were interrogated using BLASTX to search for homology to viruses within the NCBI non-redundant protein sequence database. For sequences with no or low scoring BLASTX scores (e > 10^−2^), the translated ORFs were searched for viral homologies within the non-redundant protein sequence database using DELTA-BLAST. All contigs (and minor ORFs) which remained unidentified were further searched for homologies using HHpred^46^. The identified contigs were assigned numbers C1 to Cn.

All identified viral sequences were then searched for homology, using TBLASTX, to sequences within the Expressed Sequence Tag (EST) and Transcriptome Shotgun Assembly (TSA) databases, to identify further unidentified hypothetical viruses.

### Phylogenetics

Replicase sequences (e.g. sequences containing RdRp, helicase, viral methyltransferase protein domains etc.) for homologous and out-group viruses were downloaded from GenBank. Sequence alignment was carried out using MUSCLE^47^ implemented in Geneious (6.0.6)^45^, with iterations set to 100 and clustering method set to Neighbor-joining (other settings default). Bayesian inference trees where constructed using MrBayes (2.0.6)^48^ implemented in Geneious (6.0.6)^45^. Due to the divergent nature of some of the phylogenies, where indicated, Neighbor-joining trees were constructed using Geneious tree builder (6.0.6)^45^.

### Motif Searching

MEME 4.9.1^34^ was used to search for shared motifs in 5′ and 3′ UTR sequences. The maximum number of motifs to return was set to 20 and “Search given strand only” was checked. Other settings were kept as default. Pseudoknots were predicted in both 5′UTR and 3′ UTR using DotKnot_1.3.1^49^.

### PCR Validation of Sequence Assembly

To confirm the correct assembly of each contig, PCR primer pairs were designed to generate overlapping products of 600–900 bp in length designed to cover the entire length of the contig. The size of the PCR products was assessed using gel electrophoresis and any anomalous products were Sanger sequenced by GATC Biotech (Constance, Germany).

The 5′ end of each contig was determined using 5′ RACE PCR (5′/3′ RACE Kit, 2nd Generation, Roche, Burgess Hill, UK), using the manufacturer’s protocol, followed by Sanger sequencing of the product by GATC Biotech (Constance, Germany).

### Viral Sequence Variant Analysis

Contig sequence variability was assessed by determining the number of SNPs per kb of sequence. The reads for each sample were aligned to the reference viruses separately using Bowtie2 v 2.0.5^50^. Reads were summed and genotype likelihoods calculated with samtools v1.3.1 mpileup^51^. The reference sequence for each virus was defined as the most abundant sequence present in sample 003 where possible and sample 2990 for the remaining RNAs. The variant positions were called with bcftools v1.3.1^51^, counting only variants present when the total read depth in the region was greater than 10. The number of SNPs present in each contig was summed for each sample and then the mean number of SNPs per contig was calculated.

## Electronic supplementary material

Supplementary Information
