# Supplementary material for: Multiple viral infections in Agaricus bisporus - Characterisation of 18 unique RNA viruses and 8 ORFans identified by deep sequencing
Source: Sci Rep. 2017 May 26;7:2469. doi: 10.1038/s41598-017-01592-9 (PMC5446422; doi:10.1038/s41598-017-01592-9)
Supplement: Supplementary file 1 — Supplementary Information [file 41598_2017_1592_MOESM1_ESM.pdf]

Supplementary Information:

Title: Multiple viral infections in *Agaricus bisporus* - Characterisation of 18 unique RNA viruses and 8 ORFans identified by deep sequencing

Authors: Gregory Deakin[a,b,c,1], Edward Dobbs[a,1], Ian M Jones[b], Helen M Grogan [c] , and Kerry S Burton[a, *]

Supplementary Tables

Table S1. ORFans sequenced from samples of *A. bisporus*, their RNA length and Open Reading Frame (ORF) lengths.

Name	Contig	Length	ORF Length
ORFan 1	C34	5078	513, 681, 1944
ORFan 2	C17	2311	426
ORFan 3	C19	1959	315
ORFan 4	C28	1935	315, 360
ORFan 5	C27	1110	528
ORFan 6	C38	1089	267, 276
ORFan 7	C24	927	258, 276, 324
ORFan 8	C31	703	291

The Name column corresponds to the proposed name for the discovered ORFan. The Contig column corresponds to contiguous RNA sequences assembled from the Illumina reads for each ORFan. The length and ORF length columns are in RNA bases and correspond respectively to the total length of the ORFan and length of ORFs above 250 bases.

Table S2. GenBank accession numbers for the virus and ORFan RNA molecules

Accession number	Virus name	
KY357487	Agaricus bisporus Virus 2	AbV2
KY357488	Agaricus bisporus Virus 3	AbV3
KY357489	Agaricus bisporus Virus 6 RNA1	AbV6 RNA1
KY357490	Agaricus bisporus Virus 6 RNA2	AbV6 RNA2
KY357491	Agaricus bisporus Virus 7	AbV7
KY357492	Agaricus bisporus Virus 5	AbV5
KY357493	Agaricus bisporus Virus 8	AbV8
KY357494	Agaricus bisporus Virus 9	AbV9
KY357495	Agaricus bisporus Virus 10	AbV10
KY357496	Agaricus bisporus Virus 11	AbV11
KY357497	Agaricus bisporus Virus 12	AbV12
KY357498	Agaricus bisporus Virus 13	AbV13
KY357499	Agaricus bisporus Virus 14	AbV14
KY357500	Agaricus bisporus Mitovirus 1	AbMV1
KY357501	Agaricus bisporus Virus 15	AbV15
KY357502	Agaricus bisporus Virus 16 RNA1	AbV16 RNA1
KY357503	Agaricus bisporus Virus 16 RNA2	AbV16 RNA2
KY357504	Agaricus bisporus Virus 16 RNA3	AbV16 RNA3
KY357505	Agaricus bisporus Virus 16 RNA4	AbV16 RNA4
KY357509	Agaricus bisporus Endornavirus 1	AbEV1
KY357510	Agaricus bisporus Spherical Virus	AbSV
KY357511	Mushroom Bacilliform Virus	MBV
KY357512	ORFan2	ORFan2
KY357513	ORFan3	ORFan3
KY357514	ORFan4	ORFan4
KY357515	ORFan5	ORFan5
KY357516	ORFan7	ORFan7
KY357517	ORFan8	ORFan8
KY357518	ORFan6	ORFan6
KY357519	ORFan1	ORFan1

AbV2 described above (KY357487) is the canonical variant. Additionally the Accession numbers of the two other variants discussed are:
KY357506 for the C19-C23-C1 variant of AbV2, and KY357507 for the C19-C1 variant of AbV2.

Additionally the variant of AbV6 (2990) has the Accession number KY357508.

Table S3. Abundance (depth of coverage) of the 'modular' contigs associated with the components of AbV2 (C1, C23 and C19) and their ratio in each sample/library.

	Read coverage per 1kb (FPKM)			Ratios		
Library	AbV2 (C1)	AbV2 (C23)	ORFan 3 (C19)	C1:C23	C1:C19	C23:C19
138	519.89	739.77	301.29	0.70	1.73	2.46
003-A	5350.75	5901.46	2431.71	0.91	2.2	2.43
003-B	5692.04	6534.27	3775.24	0.87	1.51	1.73
004-A	390.37	168.73	166.48	2.32	2.34	1.01
004-B	4174.26	4986.66	2304.21	0.84	1.81	2.16
1497-A	5865.43	6408.78	2993.09	0.92	1.96	2.14
1497-B	1448.05	384.89	786.84	3.76	1.84	0.49
1283	83.65	74.49	33.61	1.12	2.49	2.22
2735	482.95	658.72	246.94	0.73	1.96	2.67
2786	131.83	100.28	45.01	1.31	2.93	2.23
2919	51.82	34.10	11.94	1.52	4.34	2.86
2990	71.14	61.36	25.22	1.16	2.82	2.43
3209	264.62	363.69	114.75	0.73	2.31	3.17

Reads from each sequenced library were aligned separately to the three contigs associated with AbV2 using bowtie2 and indexed using samtools. Fragments Per Kilobase of contig per Million mapped reads (FPKM) values were calculated in order to normalise the coverage of each contig. Ratios were calculated using the FPKM values

Table S4. Viruses and their accession numbers used to construct *Hypoviridae* phylogenetic tree (Figure S1).

Accession	Short Name	Full name
M26965	PPV	*Plum pox virus*
D83408	BaMMV	*Bean mild mosaic virus*
AJ132268	BaYMV	*Barley yellow mosaic virus*
AY533037	FGDMV1	*Fusarium graminearum dsRNA mycovirus-1*
AB698498	RNV3	*Rosellinia necatrix mycovirus 3*
AF188514	CHPV3	*Cryphonectria hypovirus 3*
JF781304	SSHPV1	*Sclerotinia sclerotiorum hypovirus 1*
M57938	CHPV1	*Cryphonectria hypovirus 1*
AY307099	CHPV4	*Cryphonectria hypovirus 4*
L29010	CHPV2	*Cryphonectria hypovirus 2*
KC330231	FGHPV1	*Fusarium graminearum hypovirus 1*

The Accession column corresponds to the GenBank accession for the virus. The Short Name column corresponds to the name used in Figure S1 and the Full name column gives the taxonomic name for the virus.

Table S5. Potential regulatory regions, GC content and variation across all samples for each virus and ORFan.

Virus	5' UTR	3' UTR	Poly-A	Shared Motif	Pseudo-knots	GC%	SNPS/kb
AbV2	35	779			5	50.2	0.22
AbEV1	12	64			0	37.4	0.42
AbV3	31	47	y		0	39.8	1.27
AbV5	21	37			0	40.2	0.07
AbV6 RNA 1	30	550	y	A> 226b in 3’	3	44.9	6.18
AbV6 RNA 2	2090	378	y	A> 226b in 3’	6	42.2	4.20
AbV7	12	536	y		5	51.9	1.80
AbSV	5	345			3	45.5	5.14
AbV8	96	155			3	48.7	1.30
AbV9	32	0			0	58.3	0.17
AbV10	98	109			0	46.3	0.93
AbV11	44	164			1	45.4	2.61
AbV12	0	110			1	54.3	7.73
AbV13	50	255			2	55.0	1.09
MBV	9	250			2	46.3	3.97
AbMV1	385	183			1	35.9	0.18
AbV14	641	51	y		1	43.1	9.44
AbV15	507	105	y		0	48.2	0.10
AbV16 RNA 1	75	63	y	B> 25b in 3’	1	48.3	8.84
AbV16 RNA 2	61	53	y	B> 25b in 3’	0	50.2	10.13
AbV16 RNA 3	38	99	y	B> 25b in 3’	3	50.3	2.88
AbV16 RNA 4	47	95	y	B> 25b in 3’	0	46.2	1.79
ORFan 1	0	0				50.5	1.48
ORFan 2	1614	272				48.8	0.04
ORFan 3	668	975				48.7	0.05
ORFan 4	†	†				54.3	0.21
ORFan 5	†	†				55.4	0.27
ORFan 6	0	0				44.5	0.92
ORFan 7	†	†	y			58.7	0.32
ORFan 8	59	354	y			52.2	1.90

The 5’ UTR and 3’ UTR columns correspond to the length in bases for both of these regions. The poly-A column denotes whether a poly-A tail was associated with the sequenced virus. The shared motif column indicates a shared motif between molecules as indicated. The Pseudo-knots column corresponds to number of pseudoknots predicted in both 5’UTR and 3’ UTR using DotKnot_1.3.1 (49). The SNPS/Kb column corresponds to the mean between samples of SNPS/Kb for each virus compared to the reference sequence.
† Possibly circular

Table S6. Viruses and their accession numbers used to construct *Narnaviridae* phylogenetic tree (Figure S2).

Accession	Short Name	Full name
EU770620	EpCV	*Epirus cherry virus*
FJ157981	CsVC	*Cassava virus C*
EU770623	OuMV	*Ourmia melon virus*
JN400241	PiRV-4	*Phytophthora infestans RNA virus 4*
AF039063	ScNV-20S	*Saccharomyces cerevisiae narnavirus 20S*
U90136	ScNV-23S	*Saccharomyces 23S RNA narnavirus*
AJ004930	OMV3a	*Ophiostoma mitovirus 3a*
AM087548	OMV1a	*Ophiostoma mitovirus 1a*
AJ132755	OMV5	*Ophiostoma mitovirus 5*
AJ132756	OMV6	*Ophiostoma mitovirus 6*

The Accession column corresponds to the GenBank accession for the virus. The Short Name column corresponds to the name used in Figure S2 and the Full name column gives the taxonomic name for the virus.

Table S7. The sequences of transcripts of Brown Cap Mushroom Virus identified by Eastwood *et al.* (2015) are homologous to the AbV16 viral components and ORFan8.

Query id	Subject id	% identity	alignment length	mismatches	e-value
LN867098	AbV16 RNA 1	100	108	0	$4x10^{-58}$
LN867097	AbV16 RNA 1	97.63	169	4	$1x10^{-86}$
LN867094	AbV16 RNA 1	99.47	189	1	$8x10^{-104}$
LN867096	AbV16 RNA 2	100	99	0	$9x10^{-53}$
LN867091	AbV16 RNA 2	99.07	214	2	$3x10^{-116}$
LN867089	AbV16 RNA 2	100	237	0	$9x10^{-135}$
LN867088	AbV16 RNA 2	98.57	210	3	$4x10^{-112}$
LN867093	AbV16 RNA 3	100	98	0	$3x10^{-52}$
LN867092	AbV16 RNA 3	100	200	0	$1x10^{-112}$
LN867087	AbV16 RNA 3	99.03	414	4	0
LN867086	AbV16 RNA 4	99.79	475	1	0
LN867095	ORFan 8	100	100	0	$3x10^{-53}$
LN867090	ORFan 8	100	242	0	$1x10^{-137}$

The Query id column corresponds to the non-host transcript GenBank accession and the Subject id column corresponds to the viral segment. The % identity column indicates similarity between the aligned sequences. Alignment length corresponds to the length in nucleotides over which an alignment was found between the two sequences. The mismatches column indicates the number of mismatched nucleotides in the alignment. The e-value column indicates the likelihood of the alignment occurring due to random chance.

Table S8. Sequences and their accession numbers present in NCBI Transcriptome Shotgun Assembly database with homology to viruses of *Narnaviridae* family.

Accession	Host	Homology	e-value
HP641214	*Alasmidonta varicosa*	EpCV	$2x10^{-52}$
GACH01090825	*Chromolaena odorata*	OuMV	$3x10^{-13}$
[2]GACI01002802	*Uromyces appendiculatus*	ScNV-23s	$5x10^{-18}$
GACM01002912	*Phakopsora pachyrhizi*	PiRV-4	0
GADD01004427	*Pyropia haitanensis*	PiRV-4	$9x10^{-43}$
GAEK01031901	*Pseudotsuga menziesii var. menziesii*	CsVC	$2x10^{-9}$
GAIR01011807	*Puccinia striiformis*	ScNV-20s	$2x10^{-22}$
[2]GAIR01012025	*Puccinia striiformi*	ScNV-23s	$3x10^{-18}$
[2]GAIR01012062	*Puccinia striiformis*	ScNV-23s	$9x10^{-15}$
[2]GAIS01005902	*Puccinia striiformis*	ScNV-23s	$7x10^{-15}$
[1]KF298275	Uncultured virus isolate	ScNV-23s	$3x10^{-17}$
[1]KF298284	Uncultured virus isolate	ScNV-20s	$4x10^{-21}$

The Accession column corresponds to the GenBank accession for the homologous sequence. The Host column corresponds to the organism (if known) from which RNA was extracted. The Homology column corresponds to the virus where the homology was found and the e-value column indicates the likelihood of the alignment occurring due to random chance.

[1] Known but unnamed viruses isolated by Cook *et al*. (33)

[2] Previously reported by Cook *et al*. (33)

Table S9. Sequences and their accession numbers present in the NCBI Transcriptome Shotgun Assembly database with homology to viruses of *Tymovirales* order.

Accession	Host	Homology	e-value
GAHU01094215	*Agave Tequilana*	BOTVF	$1x10^{-22}$
GAHU01087027	*Agave Tequilana*	BOTVF	$5x10^{-23}$
GAHU01124587	*Agave Tequilana*	Pepino mosaic virus	$4x10^{-29}$
GAHU01077006	*Agave Tequilana*	BOTVF	$2x10^{-64}$

The Accession column corresponds to the GenBank accession for the homologous sequence. The Host column corresponds to the organism (if known) from which RNA was extracted. The Homology column corresponds to the virus where the homology was found and the e-value column indicates the likelihood of the alignment occurring due to random chance.

Table S10. Viruses and their accession numbers used to construct *Tymovirales* phylogenetic tree (Figure S3).

Accession	Short Name	Full name
M58152	ACLSP	*Apple chlorotic leaf spot virus*
D14995	ASGVP	*Apple stem grooving virus*
D21829	ASPVP	*Apple stem pitting virus*
AF238884	BOTVF	*Botrytis virus F*
AY055762	BOTVX	*Botrytis virus X*
AJ318061	CLBVS	*Citrus leaf blotch virus*
AJ309022	GFKVM	*Grapevine fleck virus*
X75433	GVAIS	*Grapevine virus A*
AF406744	ICRSV	*Indian citrus ringspot virus*
EU489641	LOLV	*Lolium latent virus*
AF265566	MRFVC	*Maize rayado fino virus*
D14449	PVMR	*Potato virus M*
EU835937	PVT92	*Potato virus T*
D00344	PVXX3	*Potato virus X*
M97264	SHVX	*Shallot virus X*
AY147260	SSDRV	*Sclerotinia sclerotiorum debilitation-associated virus*
X07441	TYMV	*Turnip yellow mosaic virus*
AY182001	OMSV	*Oyster musgroom spherical virus*
AF126284	AURAV	*Aura virus*
AY112987	SFV	*Semliki forest virus*
J02363	SINV	*Sindbis virus*

The Accession column corresponds to the GenBank accession for the virus. The Short Name column corresponds to the name used in Figure S3 and the Full name column gives the taxonomic name for the virus.

Ta**ble S11.** Viruses and their accession numbers used to construct the Rubi-like tree (Figure S4).

Accession	Short name	Full name
EU779934	SsRV-L	*Sclerotinia sclerotiorum RNA virus L*
AY258322	RUBV	*Rubella virus*
AY535004	AHEV	*Avian hepatitis E virus*
M73218	HEV-1	*Hepatitis E virus (Human)*
AF082843	HEV-3	*Hepatitis E virus (Swine)*
AF102884	NbetaV	*Nudaurelia capensis beta virus*
U18246	HaSV	*Helicoverpa armigera stunt virus*
AY594352	DpTV	*Dendrolimus punctatus tetravirus*
AF280539	BSBMV	*Beet soil-borne mosaic virus*
D84410	BNYVV	*Beet necrotic yellow vein virus*
AF126284	AURAV	*Aura virus*
AY112987	SFV	*Semliki forest virus*
J02363	SINV	*Sindbis virus*

The Accession column corresponds to the GenBank accession for the virus. The Short Name column corresponds to the name used in Figure S4 and the Full name column gives the taxonomic name for the virus.

Table S12. Viruses and their accession numbers used to construct alphavirus-like supergroup phylogenetic tree (Figure S5).

Accession	Virus	Grouping
AF126284	*Aura virus*	Alphavirus
AY112987	*Semliki forest virus*	Alphavirus
J02363	*Sindbis virus*	Alphavirus
	Agaricus bisporus endornavirus 1	Endornavirus
JQ951943	*Bell pepper endornavirus*	Endornavirus
JX678977	*Grapevine endophyte endornavirus*	Endornavirus
DQ399289	*Gremmeniella abietina type B RNA virus*	Endornavirus
D32136	*Oryza sativa endornavirus*	Endornavirus
AJ877914	*Phytophthora endornavirus 1*	Endornavirus
HQ380014	*Tuber aestivum endornavirus*	Endornavirus
AJ000929	*Vicia faba endornavirus*	Endornavirus
AY535004	*Avian hepatitis E virus*	Rubi-like
D84410	*Beet necrotic yellow vein virus*	Rubi-like
AF280539	*Beet soil-borne mosaic virus*	Rubi-like
AY594352	*Dendrolimus punctatus tetravirus*	Rubi-like
U18246	*Helicoverpa armigera stunt virus*	Rubi-like
M73218	*Hepatitis E virus (Human)*	Rubi-like
AF082843	*Hepatitis E virus (Swine)*	Rubi-like
AF102884	*Nudaurelia capensis beta virus*	Rubi-like
AY258322	*Rubella virus*	Rubi-like
EU779934	*Sclerotinia sclerotiorum RNA virus L*	Rubi-like
X52774	*Barley stripe mosaic virus*	Tobamo-like
X73476	*Beet yellow virus*	Tobamo-like
X01678	*Brome mosaic virus*	Tobamo-like
D00355	*Cucumber mosaic virus*	Tobamo-like
U15440	*Lettuce infectious yellow virus*	Tobamo-like
X94347	*Olive latent virus 2*	Tobamo-like
X78602	*Peanut clump virus*	Tobamo-like
AJ272328	*Pelargonium zonate spot virus*	Tobamo-like
AB000709	*Pepper mild mottle virus*	Tobamo-like
AJ238607	*Potato mop-top virus*	Tobamo-like
L07937	*Soil-borne wheat mosaic virus*	Tobamo-like
V01408	*Tobacco mosaic virus*	Tobamo-like
AF166084	*Tobacco rattle virus*	Tobamo-like
U75538	*Tobacco streak virus*	Tobamo-like
AJ132845	*Tomato mosaic virus*	Tobamo-like
KY357510	*Agaricus bisporus spherical virus*	Tymovirales
KY357497	*Agaricus bisporus virus 12*	Tymovirales
KY357498	*Agaricus bisporus virus 13*	Tymovirales
KY357502	*Agaricus bisporus virus 16*	Tymovirales
KY357488	*Agaricus bisporus virus 3*	Tymovirales
KY357492	*Agaricus bisporus virus 5*	Tymovirales
KY357489	*Agaricus bisporus virus 6*	Tymovirales

Accession	Virus	Grouping
KY357491	*Agaricus bisporus virus 7*	Tymovirales
KY357494	*Agaricus bisporus virus 9*	Tymovirales
M58152	*Apple chlorotic leaf spot virus*	Tymovirales
D14995	*Apple stem grooving virus*	Tymovirales
D21829	*Apple stem pitting virus*	Tymovirales
AF238884	*Botrytis virus F*	Tymovirales
AY055762	*Botrytis virus X*	Tymovirales
AJ318061	*Citrus leaf blotch virus*	Tymovirales
AJ309022	*Grapevine fleck virus*	Tymovirales
X75433	*Grapevine virus A*	Tymovirales
AF406744	*Indian citrus ringspot virus*	Tymovirales
EU489641	*Lolium latent virus*	Tymovirales
AF265566	*Maize rayado fino virus*	Tymovirales
AY182001	*Oyster mushroom spherical virus*	Tymovirales
D14449	*Potato virus M*	Tymovirales
EU835937	*Potato virus T*	Tymovirales
D00344	*Potato virus X*	Tymovirales
AY147260	*Sclerotinia sclerotiorum debilitation-associated virus*	Tymovirales
M97264	*Shallot virus X*	Tymovirales
X07441	*Turnip yellow mosaic virus*	Tymovirales

The Accession column corresponds to the GenBank accession for the virus. The grouping column corresponds to the phylogenetic node where the virus is located in Figure S5.

Supplementary Figures

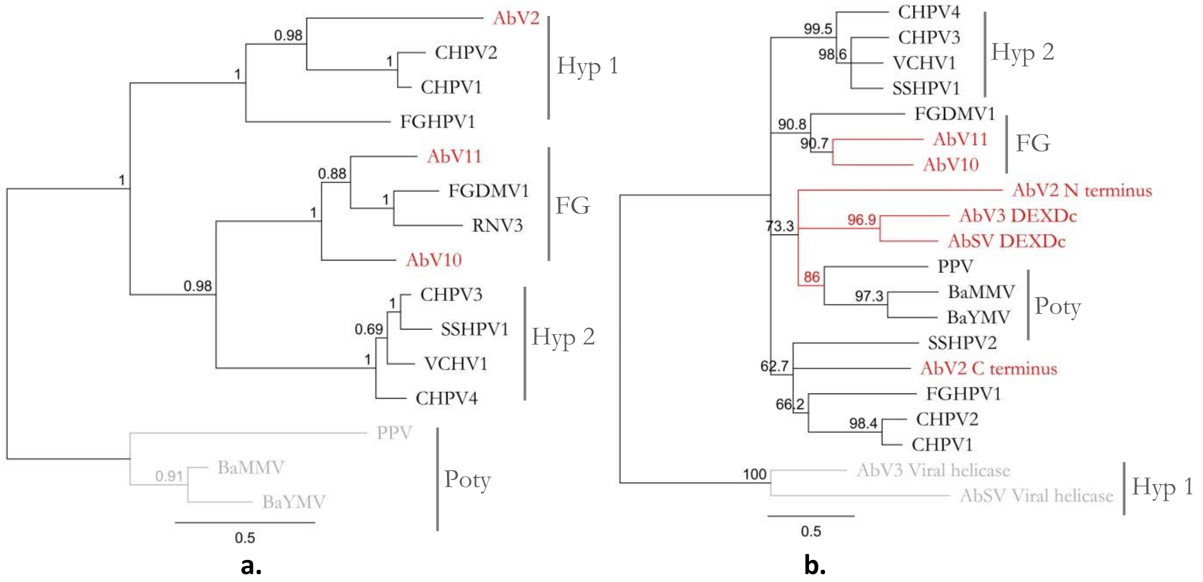

Figure S1. Comparison of proposed phylogenetic trees built using the RdRp domains and the helicase domains of the *Hypoviridae* family.

a. Bayesian inference tree of RdRp domains from selected viruses from the Hypoviridae family. The RdRp domains were aligned using MUSCLE and stripped of all gap positions. The phylogenetic tree was calculated using Mr Bayes with default parameters and a burn-in of 1,000,000 cycles. Values indicate the posterior probability of the clade. Selected members of the Potyviridae were used as an out-group.

b. Neighbour-joining tree of helicase domains from selected members of the Hypoviridae and Potyviridae families. The RNA helicase domains (SUPERFAMILY SSF52540) were aligned using MUSCLE. The Neighbour-joining tree was constructed using full alignment (no gap positions deleted). Values indicate the consensus support for the clade based on 1000 bootstrap replicates. Branches corresponding to clades with less than 60% bootstrap support were collapsed.

Red nodes indicate viruses described in this study. Grey nodes indicate out-group. Scale bars represent amino-acid substitutions per site.

Full name and accession for viruses given in Supplementary Table S4.

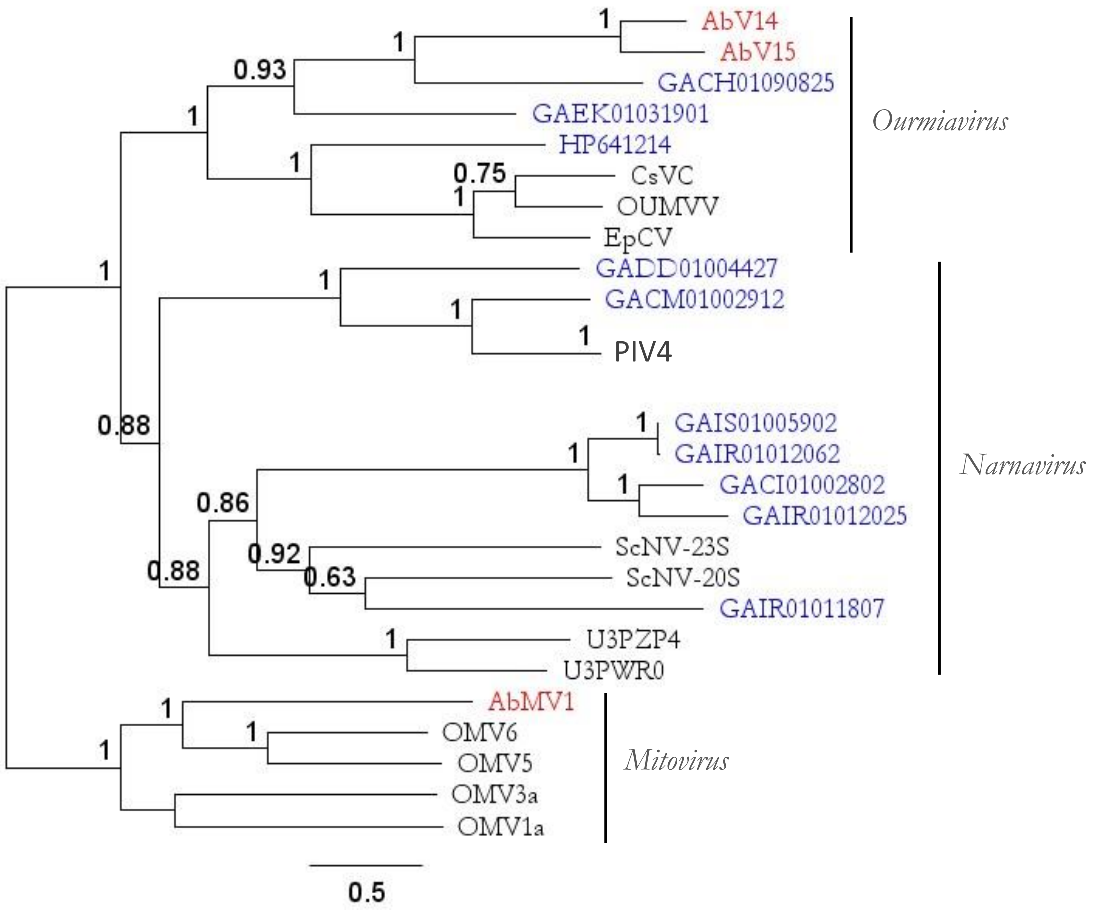

Figure S2. Proposed phylogenetic tree describing the relatedness of selected members of the *Narnaviridae* family and the *Ourmiavirus* genus. Full length replicase amino-acid sequences were aligned using MUSCLE. The Bayesian Inference Tree was calculated using Mr Bayes with default parameters and a burn-in of 1,000,000 cycles. Values indicate the posterior probability of the clade.

Red nodes indicate viruses described in this study. Blue nodes indicate viral-like sequences found in Transcriptome Shotgun Assembly database (e-values are given in Supp. Table S8). The scale bar represents amino-acid substitutions per site.

Full name and accessions for viruses given in Supplementary Table S8.

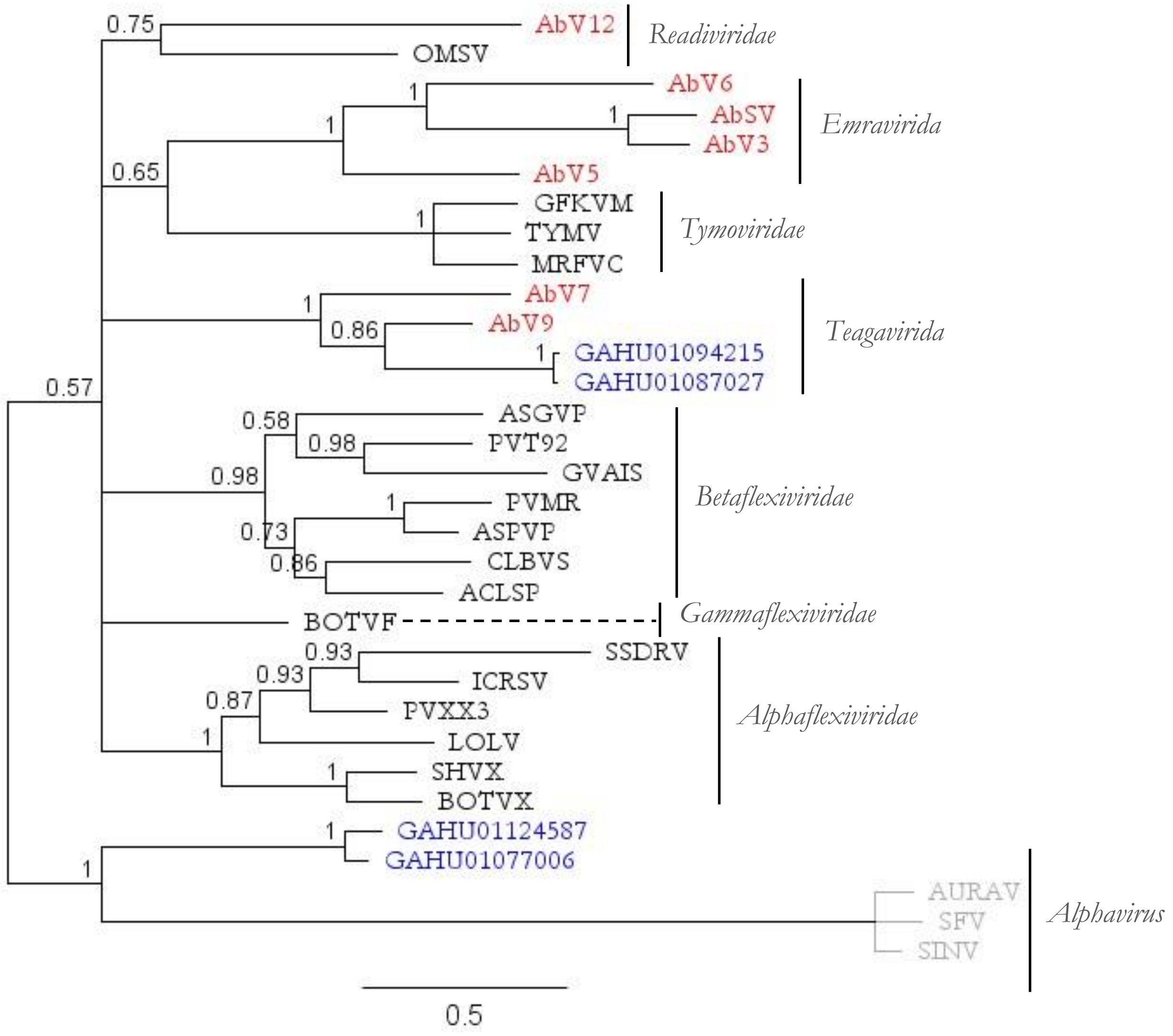

Figure S3. Proposed phylogenetic tree describing the relatedness of select viruses from the Tymovirales order. The RdRp domains were aligned using MUSCLE. The Bayesian inference tree was calculated using Mr Bayes with default parameters and a burn-in of 1,000,000 cycles. Values indicate the posterior probability of the clade. Selected members of the Alphavirus genus were used as an out-group.

Red nodes indicate viruses described here. Grey nodes indicate out-group. A search of the transcriptome database found two nearly complete viral replicase molecules in Agave tequilana (accessions GAHU01087027 and GAHU01094215) with high similarity to the C14 component (RdRp) of AbV9. Two further partial replicases similar to tymovirales replicases were also found (accessions: GAHU01077006 and GAHU01124587) (e-values are given in Supp. Table S9). These are highlighted in blue in the phylogenetic tree

The scale bar represents amino-acid substitutions per site.

Full name and accessions for viruses given in Supplementary Table S10.

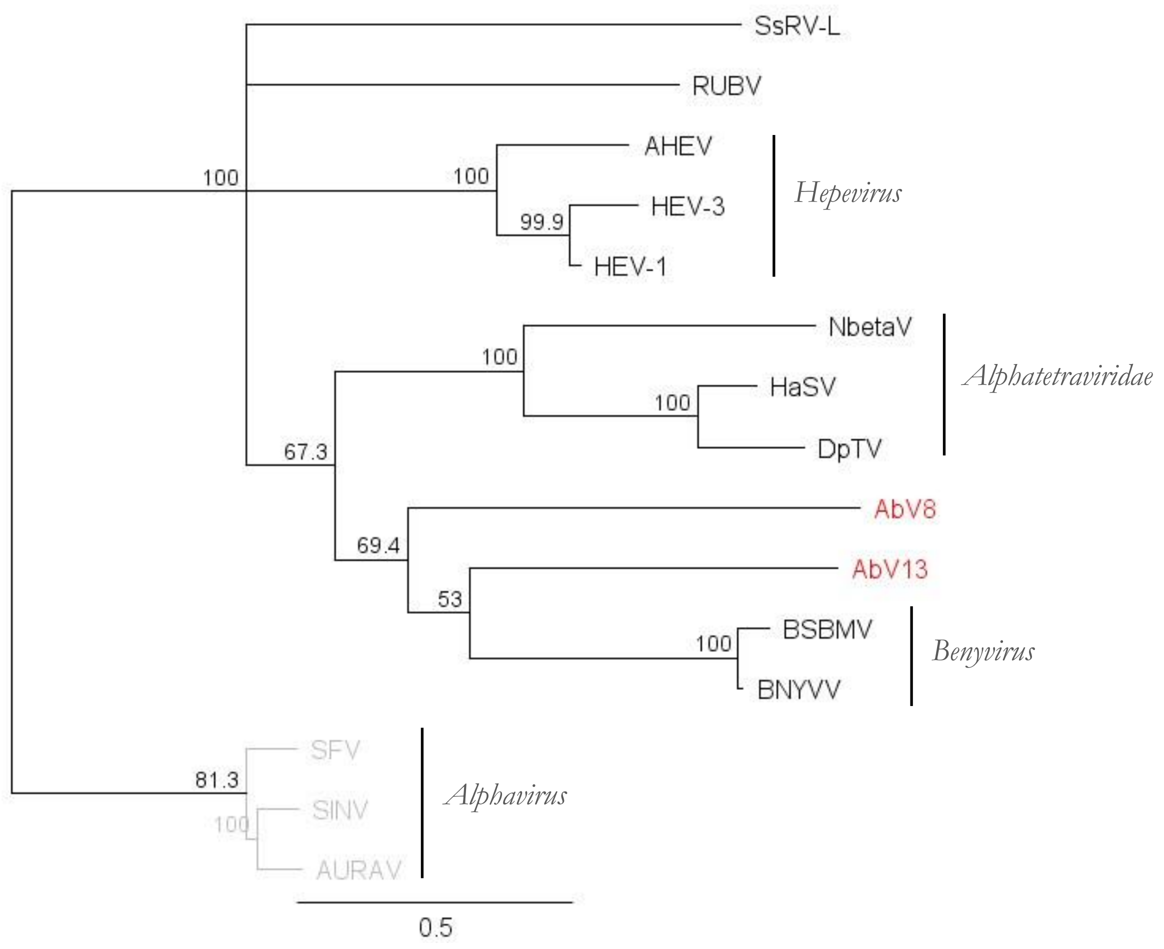

Figure S4. Proposed neighbour-joining phylogentic tree depicting the relatedness of select members of the Rubi-like viruses. The RdRp domains were aligned using MUSCLE and the phylogenetic tree calculated using a neighbour-joining algorithm implemented in Geneious. Values indicate the consensus support for the clade based on 1000 bootstrap replicates. Branches corresponding to clades with less than 50% bootstrap support were collapsed. Select members of the Alphavirus genus were used as an out-group.

Red nodes indicate viruses described here. Grey nodes indicate out-group. The scale bar represents amino-acid substitutions per site.

Full name and accessions for viruses given in Supplementary Table S11.

.

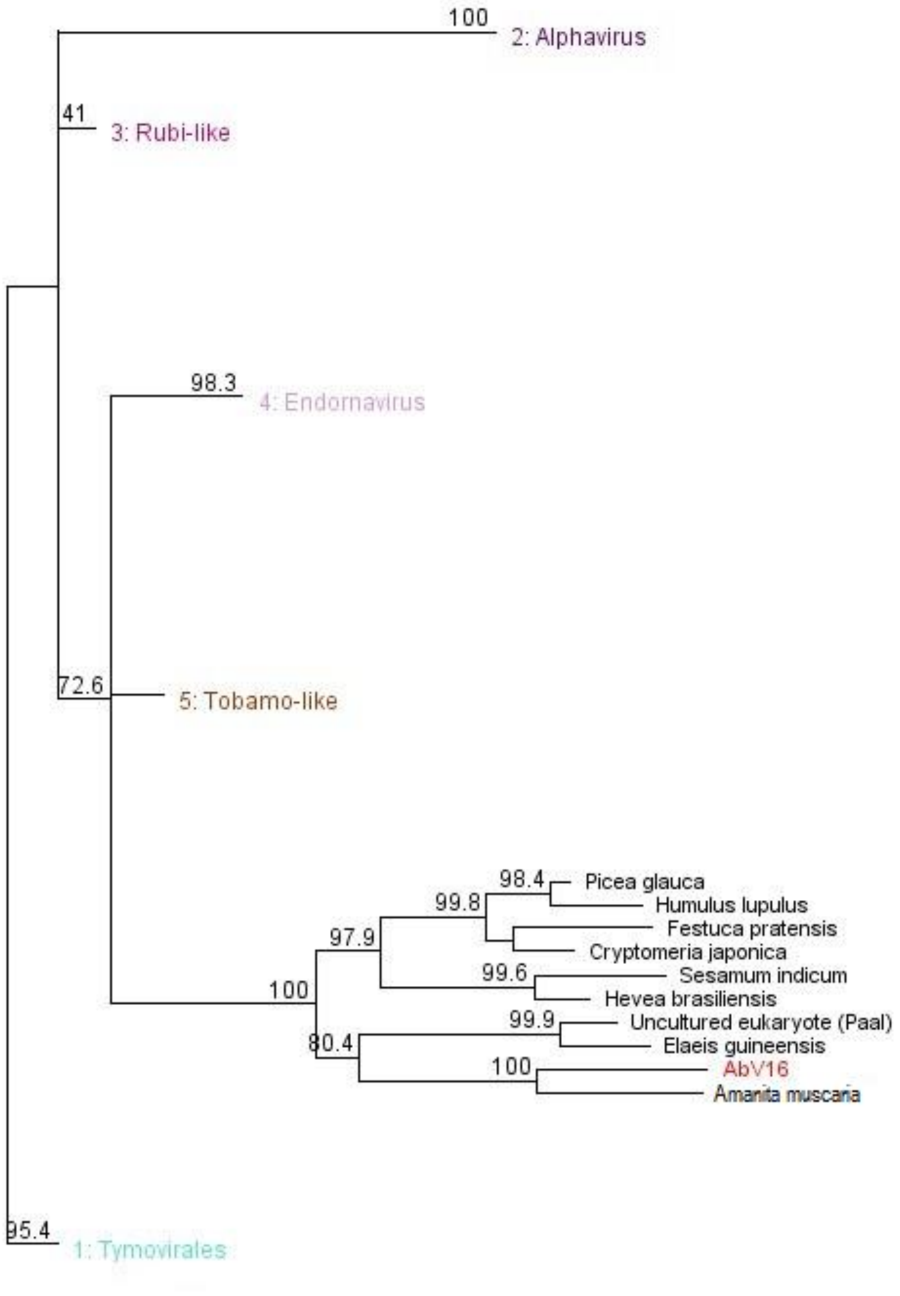

Figure S5. Proposed neighbour-joining phylogenetic tree of selected members of alphavirus-like supergroup. RdRp domains were aligned using MUSCLE and the phylogenetic tree calculated using the neighbour-joining algorithm implemented in Geneious. Branch values indicate the consensus support for the clade based on 1000 bootstrap replicates. Branches corresponding to clades with less than 40% bootstrap support were collapsed.

Numbered clades represent collapsed branches of major alphavirus-like groups. The expanded clade represents AbV16 (highlighted in red) and its homologs found in the Transcriptome Shotgun Assembly or Expressed Sequence Tag database (Table 4).

Full name and accessions for viruses used in construction of tree are given in Supplementary Table S12. Assembly or Expressed Sequence Tag database (Table 4).
